# A Novel Triplet-Primed PCR Assay to Detect the Full Range of Trinucleotide CAG Repeats in the Huntingtin Gene (*HTT*)

**DOI:** 10.3390/ijms22041689

**Published:** 2021-02-08

**Authors:** Alessandro De Luca, Annunziata Morella, Federica Consoli, Sergio Fanelli, Julie R. Thibert, Sarah Statt, Gary J. Latham, Ferdinando Squitieri

**Affiliations:** 1 Medical Genetics Division, Fondazione IRCCS Casa Sollievo della Sofferenza, 71013 San Giovanni Rotondo, Italy; a.deluca@css-mendel.it (A.D.L.); a.morella@css-mendel.it (A.M.); f.consoli@css-mendel.it (F.C.); 2 Huntington and Rare Diseases Unit, Fondazione IRCCS Casa Sollievo della Sofferenza, 71013 San Giovanni Rotondo, Italy; sergiofanelli.bio@gmail.com; 3 Asuragen, Inc., Austin, TX 78744, USA; jThibert@asuragen.com (J.R.T.); sstatt@asuragen.com (S.S.); glatham@asuragen.com (G.J.L.)

**Keywords:** Huntington disease, *HTT*-CAG repeats, novel diagnostic test, TP-PCR

## Abstract

The expanded CAG repeat number in *HTT* gene causes Huntington disease (HD), which is a severe, dominant neurodegenerative illness. The accurate determination of the expanded allele size is crucial to confirm the genetic status in symptomatic and presymptomatic at-risk subjects and avoid genetic polymorphism-related false-negative diagnoses. Precise CAG repeat number determination is critical to discriminate the cutoff between unexpanded and intermediate mutable alleles (IAs, 27–35 CAG) as well as between IAs and pathological, low-penetrance alleles (i.e., 36–39 CAG repeats), and it is also critical to detect large repeat expansions causing pediatric HD variants. We analyzed the *HTT*-CAG repeat number of 14 DNA reference materials and of a DNA collection of 43 additional samples carrying unexpanded, IAs, low and complete penetrance alleles, including large (>60 repeats) and very large (>100 repeats) expansions using a novel triplet-primed PCR-based assay, the AmplideX PCR/CE *HTT* Kit. The results demonstrate that the method accurately genotypes both normal and expanded *HTT*-CAG repeat numbers and reveals previously undisclosed and very large CAG expansions >200 repeats. We also show that this technique can improve genetic test reliability and accuracy by detecting CAG expansions in samples with sequence variations within or adjacent to the repeat tract that cause allele drop-outs or inaccuracies using other PCR methods.

## 1. Introduction

Huntington disease (HD; OMIM 143100) is an autosomal, dominantly inherited, progressive, neurodegenerative disorder caused by an expansion of a coding CAG trinucleotide repeat in the exon 1 of huntingtin (*HTT*) gene [1]. It generally manifests in adulthood, after a presymptomatic life stage of years, depending on the penetrance of the CAG mutation [2], which affects cortical–striatal connections with brain cell dysfunction and loss [3]. The main clinical manifestations appear with the unpredictable and concurrent appearance of neurological motor abnormalities (e.g., coexistence of hyper- and hypo-kinetic movement disorders, incoordination), cognitive decline (e.g., executive function abnormalities, dementia) and behavioral changes (e.g., uncontrolled emotional state, apathy, depression with suicide propensity, obsessions and perseveration, psychosis) that progressively and severely affect, altogether, the individual functional capacity until cachexia and death [4], after 15 years on average [5]. The abnormal gene product, the Huntingtin protein, is ubiquitously expressed and contains an elongated polyglutamine stretch encoded by the CAG trinucleotide repeats. Normal alleles contain up to 35 CAG repeats, whereas HD patients carry expansions of 36 or more repeats [6]. Although complete penetrance of HD is observed for CAG sizes of ≥40, only a proportion of those with a CAG repeat length of 36–39 (i.e., low penetrance alleles) shows signs or symptoms of HD within a normal life span [2,7]. Expansions of the mutation length are associated with earlier age at onset and death [5], with the first clinical manifestations occurring in young people, i.e., juvenile-onset HD (joHD), when the disease starts ≤20 years of age [8]. CAG repeat expansions beyond 60–80 trinucleotides are rare and affect children who manifest pediatric HD (PHD), with more severe disease course and an atypical phenotype compared to adults, along with reduced life span and specific neuropathological patterns [9]. However, the expanded CAG mutation penetrance, intergenerational parent–child change (i.e., CAG instability) [10], and mosaicism (i.e., cellular CAG length variability within and among the single individual tissues) [9,11,12], as well as the age at onset anticipation of offspring and the severity of disease progression, also depend, altogether, on additional factors, in addition to the CAG mutation length, including gene modifiers [13,14], loss of interruptions (LOI) in the expanded *HTT*-CAG sequence [13,15,16,17], and yet unknown environmental factors [18].

Predictive and diagnostic testing of HD require accurate sizing of the CAG repeat to distinguish the edge between normal repeats and intermediate mutable CAG alleles (IAs, 27–35 CAG) that are potentially responsible for new *HTT* mutations in offspring [2], and between the IAs and expanded mutations (i.e., beyond 35 repeats) [19]. PCR-based assays for sizing the *HTT*-CAG repeat typically involve amplification using primers flanking the CAG repeat region, followed by capillary electrophoresis (CE) [20,21]. Whenever only a single peak is detected, additional tests such as the PCR amplification of the adjacent CCG region is usually performed to exclude errors and PCR amplification failure or, in the past, Southern blot analysis to detect large expanded alleles [22,23]. The negative correlation between repeat length and amplification efficiency represents a significant deficiency of repeat-flanking PCR. Flanking sequence polymorphisms may also cause allele-specific PCR failure (allele drop-out) and lead to misdiagnosis [24,25]. In marked contrast, the more recent triplet-primed PCR (TP-PCR), a strategy that pairs a flanking primer with one that anneals randomly within the repeat to generate different-sized amplicons, produces robust amplification and reliable detection of all expanded alleles regardless of size. This is because TP-PCR products of expanded alleles generate a characteristic CE pattern that can be easily distinguished from the pattern of unexpanded alleles [26], which eliminates the need to perform the labor-intensive Southern blot methodology. In HD, the TP-PCR strategy has been used to successfully detect an expanded allele of >150 CAG repeats [27], and to detect and size an expanded allele of approximatively 180 CAG repeats [28]. Therefore, the American College of Medical Genetics and Genomics (ACMG) committee has indicated the TP-PCR as the preferred method for genetic HD testing and recommends the use of “appropriate controls that include a range of *HTT* CAG sizes”, which may be accomplished through the “use of external or internal standards” [29].

In this study, we characterized a novel TP-PCR method, the AmplideX PCR/CE *HTT* Kit, to determine assay performance and robustness. We retrospectively analyzed genetic reference materials for HD genetic testing obtained from the National Institute of General Medical Sciences Human Genetic Cell Repository at the Coriell Cell Repositories and compared them with the DNA collected from our cohort, including samples of subjects with unexpanded and expanded alleles of variable genotype and size. We also assessed the effect of sequence polymorphisms within and adjacent to the repeat tract to determine the impact, if any, on accurate genotyping using this method.

## 2. Results

**Figure 1:**
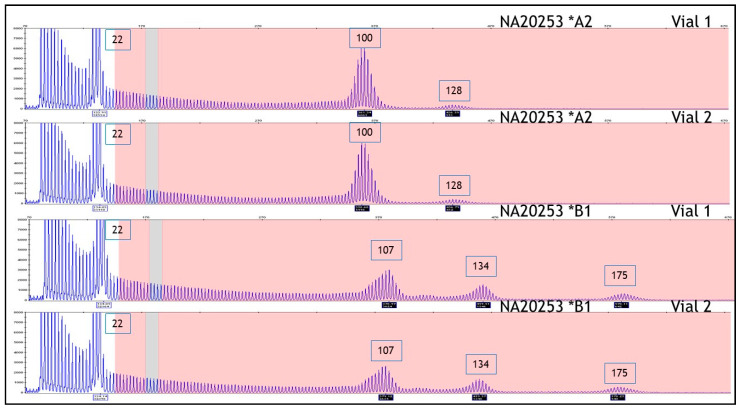
*HTT* PCR/capillary electrophoresis (CE) analysis of two lots (A2, B1) of NA20253 shows additional peaks beyond the first expanded CAG repeat peak.

**Figure 2:**
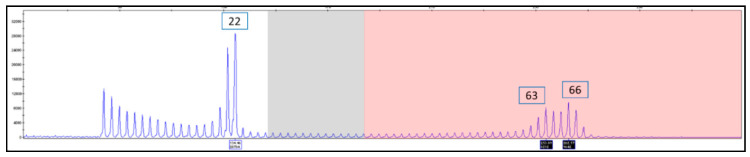
*HTT* PCR/CE analysis of NA20252 shows an additional peak (63 CAG) beyond the reference genotype of 22/66.

HD reference materials (*n* = 14) were previously determined by PCR measurement agreement across 10 laboratories and DNA sequence analysis. Allele lengths ranged from 15 to 100 CAG repeats, having the appropriate alleles to span the HD state from normal to joHD [30]. The allele(s), as identified by CAG repeat length value, and genotype were determined for each sample using the AmplideX PCR/CE *HTT* Kit. As shown in Table 1, all genotypes (as defined by categorical bin) obtained using the kit were in agreement with published genotypes across all cell line samples examined, with only one cell line DNA showing a single CAG repeat difference from the expected repeat length. This lone deviation, sample NA20210, has a reference heterozygous compound genotype of 17 and 74 CAG repeats, although we observed 17 and 75 repeats (see Table 1 for details). However, this difference lies well within error limits recommended by both ACMG [29] and EMQN [31] and, furthermore, the sample was reported as 17 and 75 repeats by DNA sequencing [30]. Moreover, in sample NA20253, a 3rd CAG repeat peak at 128 CAGs was repeatedly detected in the electropherogram in addition to the expected 22 and 100 CAG repeats, which were both correctly identified. These results are in accordance with an external investigation of this cell line that revealed the existence of this 3^rd^ peak and possibly a 4th peak across two Coriell cell-line lots (Figure 1, compare A2 & B1). The 3^rd^ peak of 128 repeats in lot A2 shifted to 134 repeats in lot B1, while also becoming more prominent; a 4th peak of 175 repeats appeared in lot B1 only. The primary expanded peak of 100 CAGs also shifted in B1 to 107 repeats, whereas the unexpanded peak remained unchanged at 22 repeats. Lot B1 was a subsequent passage of the parent cell line, and thus, the shift in the large expanded alleles likely reflects mutations that increased in length with passage of the cell line in this unstable region of the *HTT* gene. Similarly, in sample NA20252, a 3^rd^ CAG repeat peak was identified at 63 CAGs in the electropherogram that was not reported in the Kalman study [30] (Figure 2).

**Figure 3:**
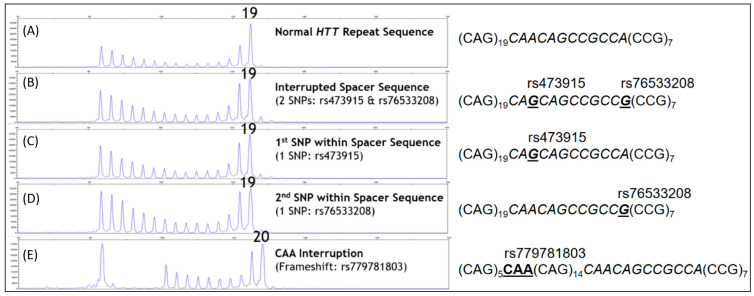
Known polymorphisms adjacent to or within the CAG repeat tract. The AmplideX PCR/CE *HTT* Kit accurately genotypes templates with known polymorphisms adjacent to or within the CAG repeat tract. (**A**) Template with 19 repeats showing the expected CE electropherogram and corresponding genotype for the relevant portion of the *HTT* reference sequence. (**B**) DNA with two SNPs (rs473915 A>G and rs76533208 A>G) interrupting the 12-bp spacer region (shown in italics) is successfully amplified to produce the expected 19 CAG result. (**C**,**D**) PCR/CE genotyping of each SNP (rs473915 or rs76533208, respectively) results in the correct 19 CAG call. (**E**) A CAA frameshift (rs779781803) within the repeat sequence shows a repeat-primed amplification pattern and a terminal peak corresponding to 20 CAG, rather than 19 CAG, due to the mobility shift caused by the triplet interruption. Note that the CAA interruption causes a signal “dip” in the repeat-primed pattern; this dip flags result as suspicious for unexpected sequence variation.

Having demonstrated the assay accuracy with HD reference materials, we next assessed the robustness of the kit to polymorphisms adjacent to and within the CAG repeat tract. As a first step, synthetic templates with 19 CAG repeats and three well-established polymorphisms (rs473915, rs76533208, and rs779781803) were amplified either alone or in combination at copy-number inputs comparable to that of the *HTT* gene when using genomic DNA. Both rs473915 and rs76533208 are loss-of-interruption A-to-G SNPs with profound impacts on HD age of onset [15,16,17]. They are also known to cause allele drop-outs in PCR [24,25]. By comparison, the rs779781803 variant interrupts the CAG tract with a CAA sequence. As shown in Figure 3, templates bearing all three polymorphisms, including one with rs473915 and rs76533208 on the same molecule, were robustly amplified and genotyped at or within one repeat of the correct value. We further evaluated 10 other sequence variations immediately downstream of the repeat segment that were most prevalent among *HTT* alleles sequenced elsewhere (Tables S1 and S2) [13]. Templates with each of these variations were also readily amplified in the PCR/CE kit assay, resulting in genotypes that were within a single repeat number of the correct value except for three samples that had deletions within the canonical CAACAGCCGCCA spacer sequence and shifted the amplicon CE mobility accordingly (Tables S1 and S2 and Figure S1).

**Figure 4:**
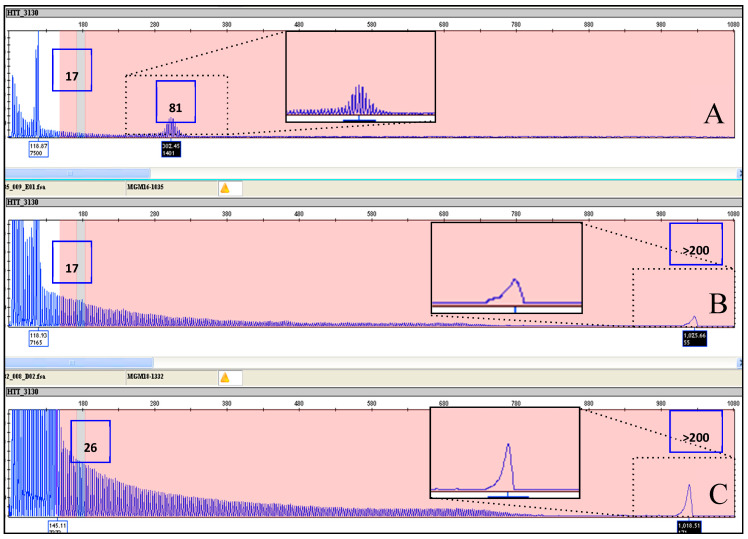
Electropherogram results for samples with unusually large CAG expansions. (**A**) Electable 16. showing alleles of 17/81 CAG repeats and a continuous stuttering after the first prominent peak. (**B**,**C**) show sample MGM16-1035 and sample MGM18-1332, respectively, with the presence of a pile-up peak at the end of the run representing the high CAG expansion of 200 repeats (zoom of pile-up in **B**,**C**).

Our next step was to analyze a collection of 43 DNA samples, provided by LIRH Foundation to the Huntington and Rare Diseases Unit of CSS-Mendel Institute, section in Rome, Italy, of IRCCS Casa Sollievo della Sofferenza Research Hospital. These included 2 samples with alleles in the normal range (6–26 CAGs), 2 samples with IAs (27–35 CAGs), 6 samples with incomplete penetrance alleles (36–39 CAGs), and 33 samples carrying alleles with full penetrance (>40 CAGs). Of the alleles with full penetrance, 20 were from joHD/PHD patients carrying CAG expansions larger than 60 repeats. Of these, 41 samples had been previously tested in our laboratory using either fluorescent repeat-flanking PCR or an alternative triplet-primed PCR method, or both, whereas 4 samples had been tested in other HD reference laboratories. Primer sequences and reaction conditions of the fluorescent repeat-flanking PCR method and of the tripled-primed PCR method are described in the methods section. Similar to Coriell cell lines, the genotypes obtained using the AmplideX PCR/CE *HTT* Kit were identical, or with discrepancies comprised within the acceptable error limits established by the HD genetic testing guidelines [29,31], also in this series (see Table 2 for details). The assay accurately sized 18 samples with very large alleles comprised between 60 and 100 CAG repeats, and it detected two exceptionally large alleles with more than 200 CAG repeats (Table 2 and Figure 4A). In particular, samples MGM16-1035 and MGM18-1332, which could not be detected by standard fluorescent PCR, were clearly identified by an AmplideX PCR/CE *HTT* Kit and determined to have an expanded allele of >200 CAG repeats (Figure 4B,C). Nevertheless, only a stuttering pattern with a continuous repeat peak pattern and a pile-up peak at the end of the run was seen, since the upper limit for accurately size-expanded alleles is less of 200 CAG repeats.

**Figure 5:**
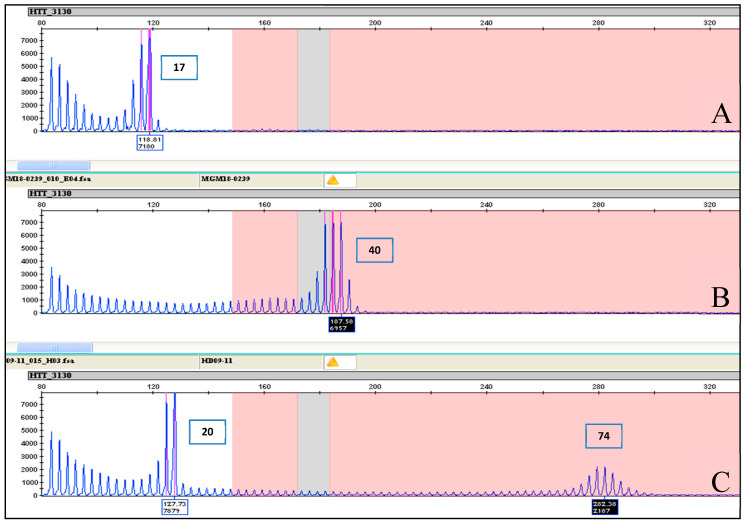
Difference between homo- and heterozygous alleles electropherogram results. (**A**) Electropherogram results of sample MGM17-1760 showing a homozygous allele of 17 CAG repeats. (**B**) Electropherogram results of sample MGM18-0239 showing a homozygous allele of 40 CAG repeats. (**C**) Electropherogram results of sample HD09-11 showing a heterozygous allele of 20 CAG repeats and an expanded allele of 74 CAG repeats. Note the continuous stuttering after the prominent peak at 20 CAG repeats to peak at 74 CAG repeat in the close-up view, which is an indication of the presence of an expanded allele.

Samples with true homozygous normal genotypes were distinguished from heterozygote samples with expanded alleles by the absence of a stuttering pattern denoting an expanded allele (Figure 5A,B). As expected, an electrophoretic pattern overlapping that of normal homozygotes was observed in the case of extremely rare genotypes with double expanded alleles (Figure 5C).

## 3. Discussion

With the improved clinical and genetic knowledge of neurodegenerative diseases, the accuracy and sensitivity of genetic testing technologies is strongly required to confirm the wide spectrum of heterogeneous clinical manifestations. Reliable genetic tests help to properly stratify patients and possibly address them to clinical research trials with the potentiality of disease modification, which are emerging in recent years.

Concerning HD, the accurate sizing of the CAG repeat number, throughput, efficiency, and inter-laboratory accuracy are cornerstones for genetic testing. In the present study, we evaluated the accuracy of the AmplideX PCR/CE HTT Kit in sizing the number of CAG repeats in the HTT gene. As for the other protocols based on TP-PCR, this assay provides an electrophoretic pattern characterized by a continuous stuttering of peaks representing CAG expansion after the first prominent peak (see Figure 1, Figure 2, Figure 3, Figure 4 and Figure 5 for details). For this reason, this method easily distinguishes between a healthy homozygote (i.e., an individual carrying two unexpanded alleles of identical size) and heterozygotes with large expanded alleles that could be hard to distinguish with other PCR-based methods and confused with healthy homozygotes. In HD laboratory testing, it is extremely important to precisely discriminate the CAG repeat variability falling in the edge between the normal and the IA range, and those falling between the IA range and pathological low penetrance range of triplets. In these cases, the difference of a few CAG repeats may represent a burden for genetic diagnosis, mainly when the genetic test result is addressed to presymptomatic, at-risk people. In this study, all our samples were genotyped precisely, demonstrating the accuracy of this method for diagnosing HD. The very few discrepancies in genotyping we observed with respect to the reference samples were perhaps due to the use of different size standards in CE runs—e.g., Asuragen assay uses ABI ROX1000 and “in house” fluorescent repeat-flanking PCR assay uses ABI ROX500—and were for highly expanded and mosaic alleles and, anyhow, within the tolerated error according to the international EMQN/CMGS [31] and ACMG [29] guidelines for HD genetic testing, allowing us to assign each sample to its correct genotypic class (see Table 1 and Table 2 for details).

Compared to the other published systems, this method has also the advantage of allowing the accurate genotyping of allelic expansions up to hundreds of CAG repeats, therefore permitting the accurate genotyping of challenging cases such as those with large expansions that are typical of the joHD form. Furthermore, as demonstrated for the first time in this study, this method allows the safe identification of allelic expansions with more than 200 repeats, which are present in the rarest PHD form. In these cases, even if it is not possible to calculate the accurate number of triplets, the method clearly identified the expanded allele, which appears as a pile-up peak at the end of the run. As expected, the system was also capable of correctly genotyping expanded homozygous genotypes, which are visible as a single expanded peak in the full mutation range with the absence of a peak in the normal range. Finally, this method also correctly genotyped cell-line reference materials that are used as size references for HD molecular diagnosis and identified previously undetected extended expanded mosaic alleles (see Figure 1 and Figure 2) as additional peaks in the electropherograms of previously published samples [30].

In terms of cost, time, and effort, compared to other apparently less expensive methods, such as fluorescent repeat-flanking PCR, the AmplideX PCR/CE *HTT* Kit has the advantage of simplifying the testing algorithm by lowering, if not eliminating, the number of samples needing further testing by CGG or CGG+CAG, and thus reducing the time and effort spent in testing. Other *HTT* screening methods based on TP-PCR have also been developed, such as TP-PCR melting curve analysis [32]. While being cost-effective and amenable to high throughput, TP-PCR melting curve analysis is not able to determine the exact allele sizes in a DNA sample. This aspect represents an important limitation in the clinical setting, because all expansion-positive samples (HD-affected), as well as those samples within the cutoffs between unexpanded and intermediate alleles, or between IAs and pathological, low-penetrance alleles, should be followed up with sizing confirmation.

There is a need to standardize and improve the accuracy, sensitivity, and the specificity of the genetic determination of the precise expanded trinucleotide number within different populations for diagnostic purposes [33]. This is particularly true in light of potential results of new, upcoming, disease-modifying and neuroprotective therapies that could be used to treat people at the very first HD or presymptomatic life stages in the future.

These results also demonstrate the utility of the assay for research purposes. For example, CAG repeat mosaicism, a phenomenon occurring in dynamic expanded mutations such as in HD, represents an important research topic with implication with clinics [12,15,16], mainly in case of large size alleles [9]. Thus, genetic labs committed to research on HD may benefit from careful discrimination of mosaicism detection for research purposes in the future. Another relevant example is related to the clinical trials recently aiming to test experimental antisense oligonucleotide therapies that recommended precise CAG number and patient’s age in a math formula for inclusion criteria determination. Finally, there is strong need to raise awareness among families with at-risk children by the global HD community (e.g., pharma, families, and researchers) after the European Medicines Agency (EMA) removed a class waiver that allowed sponsors to exclude children and adolescents from clinical studies for a number of conditions, including HD, and recommended a Pediatric Investigation Plan (PIP) for future therapeutic trials [34]. Such recommendations aim to extend medicines following future positive trial results to minors, who are still excluded from all clinical experimental therapies. Therefore, the first step is to approach such a young population and their parents through careful counselling [35] by a clinical diagnosis that needs to be confirmed by a reliable genetic test, whenever allowed [36]. For instance, the methodology we describe here contributes to reliably detect highly expanded mutations above 200 CAG repeats thanks to the visible smear between the normal and the pathological alleles.

We are aware that new upcoming technologies aiming to detect intra-CAG repeat patterns [15,16,17] or bioinformatics tools [37] will help improve the genotyping of repeat-expansion alleles in the future. However, a highly qualitative vs. quantitative, PCR-based measurement of the triplet number for a wide delivery by genetic lab services is urgently needed. Therefore, the methodology we describe might help standardize HD diagnosis among genetic laboratories, with implications for genetic pathologies other than HD, e.g., Fragile X (FMR1 gene) [38], ALS/FTD (C9orf72 gene) [39], and Myotonic Dystrophy Type I (DMPK gene) [40], while providing the sensitivity and versatility to detect a broader spectrum of allelic conditions within HD itself. Remarkably, this assay was shown to successfully amplify and accurately genotype DNA harboring rare polymorphisms near the CAG repeat region that can cause allelic drop-outs, thus mitigating, if not eliminating, false-negative results that challenge current HTT PCR technologies [41]. The improved sensitivity is particularly needed in cases of presymptomatic HD, where normal allele homozygosity may rarely mask the expanded allele amplification [24,25]. By reducing such risks, more definitive results are possible from the initial test with the potential for diagnostic labs to shorten turn-around times by minimizing complex follow-on testing, such as allele sequencing.

## 4. Materials and Methods

### 4.1. Samples

Study cohorts included (1) DNA samples of a set of 14 HD cell lines with associated genotype data available from Coriell Cell Repositories (Camden, NJ, USA) that are used as reference materials for *HTT* CAG repeat sizing [30], and (2) DNA samples of a set of 43 additional subjects available from LIRH Foundation and genetically characterized at CSS-Mendel Institute, section in Rome, Italy, of IRCCS Casa Sollievo della Sofferenza Research Hospital (San Giovanni Rorondo, Italy). The Coriell HD reference samples included 4 cases with alleles within the normal range, 1 case with IA, 2 cases with alleles with low penetrance, and 7 samples carrying alleles with complete penetrance. Of the samples with alleles with complete penetrance, 2 harbored alleles larger than 60 CAG repeats, which are generally seen in subjects with joHD and in the rarest PHD variants (Table 1). Our 43 DNA sample collection included 2 cases with alleles within the normal range, 2 cases with IA, 6 with low penetrance, and 33 with complete penetrance. Of the samples with alleles with complete penetrance, 20 harbored alleles larger than 60 CAG repeats, which are generally seen in subjects with joHD and in the rarest PHD variants. Finally, we also included 2 samples from subjects homozygous for CAG repeat expansion (Table 2). These 43 samples had been previously tested in our laboratory using an “in-house” fluorescent repeat-flanking PCR (primer sequences and PCR protocols for this method are available upon request), whereas four had been tested in other HD reference laboratories (Table 2). The HD alleles in the 14 HD cell lines ranged in size from 15 to 100 CAG repeats (refer to [30] for details on cell-line genotypes). The HD alleles from our collection ranged from 37 to >200 CAG repeats.

### 4.2. AmplideX PCR/CE HTT Kit

The AmplideX PCR/CE *HTT* Kit (cat#: 49657; Asuragen, Inc., Austin, TX, USA) was used to PCR amplify the *HTT* trinucleotide CAG fragment starting from 20 ng total of purified genomic DNA, isolated from peripheral blood leukocytes using the Gentra Puregene Blood Kit (Qiagen, Germantown, MD, USA), or the MagCore Genomic DNA Whole Blood Kit (Diatech Lab line, Jesi, Italy). Samples were prepared for the PCR with a master mix from Asuragen containing *HTT* PCR Mix (5.0 uL), *HTT* forward, reverse Primer Mix (3.0 uL), and an internal calibrator. Aliquots of the DNA sample, typically 2 uL at 10 ng/uL, and 8 uL master mix were vortex-mixed, centrifuged, and transferred to a thermal cycler. Thermal cycling was performed on Applied Biosystems’ Veriti and 9700 Thermal Cyclers (Applied Biosystems, Foster City, CA, USA) using the following cycle: 95° C for 5 min, 10 cycles of 97° C for 35 s, 64° C for 35 s, 68° C for 4 min, 18 cycles of 97° C for 35 s, 64° C for 35 s, 68° C for 4 min plus 20 sec/cycle, and final extension at 72° C for 10 min, 4° C hold. Then, 2 uL of PCR product was mixed with 11 uL Hi-Di Formamide (Applied Biosystems, Foster City, CA, USA) and 2 uL ROX 1000^TM^ Size Ladder (Asuragen, Inc., Austin, TX, USA). Samples thus prepared were denatured at 95° C for 2 min and then cooled at 4° C and held until ready for analysis by capillary electrophoresis (3130xL Genetic Analyzer, Applied Biosystems, Foster City, CA, USA). The FAM-labeled amplicons were detected using the following fragment analysis protocol: 36cm capillary, 2.5 kV, 20 s injection, and 15 kV run for 2400 s. Capillary electrophoresis run parameters were adjusted to extend POP-7 sizing range beyond 200 repeats. Lower run voltages (2.5 kV, 40 s injection) were used to allow the identification of hyper-expanded allele over to 200 repeats, with an increase in run time and loss in sensitivity due to the spreading of the signal within a “pile-up peak” (>200 repeats). This peak comprises aggregated amplicons too long to be adequately resolved by CE electropherograms, which were processed as .fsa files using GeneMapper v4.1 for analysis and manually annotated for repeat profile initiation and the size of the full-length product. A PCR control admixture sample comprising *HTT* alleles with 17, 39, 50, and 75 repeats and a no-template control were used in all experiments. Data analysis and interpretation was conducted using the fragment analysis software GeneMapper v4.1 and an Excel-based analysis tool, AmplideX PCR/CE *HTT* Macro (Asuragen, Inc., Austin, TX, USA). The AmplideX PCR/CE *HTT* Kit Macro can determine size and mobility correction, repeat size was determined using a linear fit adjustment of the ROX ladder size peaks to the PCR/CE control sample alleles. Alleles are reported as integer CAG repeats. The largest allele size determines genotype category: normal, intermediate, reduced penetrance or expanded. The alleles up to 200 CAG repeats are reported; the alleles >200 repeats are identified as “> 200”.

To determine the effects of polymorphisms in and near the repeat tract, ultramer DNA oligonucleotides (Integrated DNA Technologies, Coralville, IA, USA) were synthesized as controls. Ultramer samples were input at 10,000 copies and amplified on the Veriti Thermal Cycler using the AmplideX PCR/CE *HTT* Kit following the manufacturer’s protocol guide. Post-PCR allelic detection of the fluorescently-labeled products were resolved by capillary electrophoresis on the 3500xL Genetic Analyzer (Applied Biosystems, Foster City, CA, USA) with a 50cm capillary with the fragment analysis protocol as described above, except that a 19.5 kV run was performed. Genotypes were determined from the mobility of the target amplicon in combination with the repeat-primed peak pattern.

## 5. Conclusions

In conclusion, the AmplideX PCR/CE *HTT* Kit provides a rapid, sensitive, and reliable method to accurately genotype the *HTT* CAG repeat region while extending the detection limit of expanded alleles to over 200 CAG repeats. Thus, this methodology provides a comprehensive molecular diagnostic evaluation to detect the full range of *HTT*-CAG trinucleotides of all HD subjects, including pediatric forms carrying extremely large, hard-to-detect alleles and avoiding the use of Southern analysis to estimate the size. Finally, the AmplideX PCR technique provides an accurate approach to easily and rapidly detect mutations in those cases where particular nucleotide polymorphisms may erroneously generate false-negative results. A missing genetic diagnosis may have critical implications, both for individuals carrying the risk of HD and their families.

## Figures and Tables

**Table 1:** Huntington disease (HD) reference samples with published allele sizes [30] compared to those determined using the AmplideX PCR/CE *HTT* Kit.

Vendor ID	Input (ng)	Categorical Bin	Published (Kalman et al., 2007)		Measured AmplideX PCR/CE *HTT* Kit	
			Allele 1	Allele 2	Allele 1	Allele 2
NA20245	20	Normal	15	15	15	15
NA20206	20	Normal	17	18	17	18
NA20207	20	Normal	19	21	19	21
NA20246	20	Normal	15	24	15	24
NA20247	20	Intermediate	15	29	15	29
NA20248	20	Reduced Penetrance	17	36	17	36
NA20249	20	Reduced Penetrance	22	39	22	39
NA20208	20	Expanded	35	45	35	45
NA20209	20	Expanded	45	47	45	47
**NA20210**	**20**	**Expanded**	**17**	**74**	**17**	**75**
NA20250	20	Expanded	15	40	15	40
NA20251	20	Expanded	39	50	39	50
NA20252	20	Expanded	22	65 & 66	22	66 *
NA20253	20	Expanded	22	100	22	100 **

Allele genotypes for Coriell samples shown from Kalman et al. [30] are the mode; error limits were set at ±1 for alleles ≤42 repeats and ±3 repeats for alleles >42 CAG repeats, according to the European Molecular Genetics Quality Network best practice guidelines for the molecular genetic testing of Huntington disease [31]. * A 63 CAG mosaic allele was also detected. ** A 128 CAG mosaic allele was also detected. Underlined and in bold, the difference between the two methodologies.

**Table 2:** HD samples used for this validation and corresponding genotypes obtained using fluorescent repeat-flanking PCR.

LAB ID	Categorical Bin	“In House” PCR		AmplideX PCR/CE *HTT* Kit	
		Allele 1	Allele 2	Allele 1	Allele 2
MGM17-1760	Normal	17	17	17	17
MGM18-1250	Normal	17	17	17	17
MGM17-0052 *	Intermediate	15	29	15	29
MGM17-0089	Intermediate	19	32	19	32
MGM16-0689	Reduced penetrance	17	37	17	37
HD157-02	Reduced penetrance	17	37	17	37
MGM16-0828	Reduced penetrance	17	37	17	37
MGM17-1738	Reduced penetrance	17	39	17	39
MGM15-1651	Reduced penetrance	24	39	24	39
HD23-05	Reduced penetrance	33	39	33	39
MGM16-0607	Full penetrance	20	40	20	40
MGM18-0177	Full penetrance	14	41	14	41
CTRL-DNA_S0162119 *	Full penetrance	17	41	17	41
MGM16-0196	Full penetrance	17	41	17	41
MGM18-0239	Full penetrance	40	40	40	40
CTRL-DNA_S0162120 *	Full penetrance	15	42	15	42
MGM16-0086	Full penetrance	18	42	18	42
MGM17-1838	Full penetrance	17	43	17	43
HD672-01	Full penetrance	17	44	17	44
MGM16-0250	Full penetrance	17	46	16	46
MGM18-0181	Full penetrance	40	47	40	47
HD423-02	Full penetrance	22	50	22	50
HD09-08	Full penetrance	18	59	18	59
HD87-07	Full penetrance	17	62	17	62
MGM17-1716	Full penetrance	17	63	17	63
MGM17-0054 *	Full penetrance	22	63	22	63
MGM18-0054	Full penetrance	22	63	22	63
MGM18_0054	Full penetrance	22	63	22	63
HD438-01	Full penetrance	17	66	17	66
MGM17-1387	Full penetrance	20	74	20	74
HD09-11	Full penetrance	20	74	20	74
MGM16-1030	Full penetrance	26	76	26	76
HD315-01	Full penetrance	26	78	26	78
**MGM16-1033**	**Full penetrance**	**17**	**80**	**17**	**81**
**MGM16-1032**	**Full penetrance**	**33**	**86**	**33**	**87**
HD636-01	Full penetrance	17	86	17	86
HD09-12	Full penetrance	18	86	18	86
**MGM16-1031**	**Full penetrance**	**19**	**88**	**19**	**89**
HD379-05	Full penetrance	18	89	18	89
MGM16-1034	Full penetrance	18	96	18	96
HD130-05	Full penetrance	25	98	25	98
**MGM16-1035**	**Full penetrance**	**17**	**84**	**17**	**>200**
**MGM18-1332**	**Full penetrance**	**26**	**95**	**26**	**>200**

Acceptable error limits are ±1 repeat for alleles ≤42 and ±3 repeats for alleles >42 [31]. * Samples tested in other HD reference laboratories. Underlined and in bold is the difference between the two methodologies.

## Data Availability

The data that support the findings of this study are available from the corresponding author upon reasonable request.

## Supplementary Materials

Supplementary Materials can be found at https://www.mdpi.com/1422-0067/22/4/1689/s1.
