# Supplementary material for: A Novel Triplet-Primed PCR Assay to Detect the Full Range of Trinucleotide CAG Repeats in the Huntingtin Gene (HTT)
Source: Int J Mol Sci. 2021 Feb 8;22(4):1689. doi: 10.3390/ijms22041689 (PMC7916029; doi:10.3390/ijms22041689)

Supplemental File for De Luca et al.

Table S1. Sequence and prevalence of canonical and non-canonical polymorphisms as detailed by GeM-HD Consortium et al., Cell (2019): 178, 887-900.

Canonical single CAA interruption	HD Expanded (>35 CAG) chromosome (number)	HD Expanded (>35 CAG) chromosome (frequency)	HD Normal (<36 CAG) chromosome (number)	HD Normal (<36 CAG) chromosome (frequency)	Non-HD Expanded (>35 CAG) chromosome (number)	Non-HD Expanded (>35 CAG) chromosome (frequency)	Non-HD Normal (<36 CAG) chromosome (number)	Non-HD Normal (<36 CAG) chromosome (frequency)
CAA1 CAG1 CCG1 CCA1 CCG7 CCT2 CAGCTTCCT1	789	81.09%	552	54.82%	12	92.31%	5,121	54.12%
CAA1 CAG1 CCG1 CCA1 CCG10 CCT2 CAGCTTCCT1	120	12.33%	311	30.88%	1	7.69%	3,055	32.28%
CAA1 CAG1 CCG9 CCT2 CAGCTTCCT1	6	0.62%	57	5.66%	0	0.00%	412	4.35%
CAA1 CAG1 CCG1 CCA1 CCG9 CCT2 CAGCTTCCT1	8	0.82%	23	2.28%	0	0.00%	407	4.30%
CAA1 CAG1 CCG1 CCA1 CCG9 CCT3 CAGCTTCCT1	1	0.10%	12	1.19%	0	0.00%	131	1.38%
CAA1 CAG1 CCG1 CCA1 CCG6 CCT2 CAGCTTCCT1	1	0.10%	5	0.50%	0	0.00%	12	0.13%

Non-Canonical	HD Expanded (>35 CAG) chromosome (number)	HD Expanded (>35 CAG) chromosome (frequency)	HD Normal (<36 CAG) chromosome (number)	HD Normal (<36 CAG) chromosome (frequency)	Non-HD Expanded (>35 CAG) chromosome (number)	Non-HD Expanded (>35 CAG) chromosome (frequency)	Non-HD Normal (<36 CAG) chromosome (number)	Non-HD Normal (<36 CAG) chromosome (frequency)
CAA1 CAG1 CAA1 CAG1 CCG1 CCA1 CCG7 CCT3 CAGCTTCCT1	17	1.75%	42	4.17%	0	0.00%	261	2.76%
CCG1 CCA1 CCG7 CCT2 CAGCTTCCT1	8	0.82%	1	0.10%	0	0.00%	7	0.07%
CCG12 CCT2 CAGCTTCCT1	9	0.92%	0	0.00%	0	0.00%	2	0.02%
CCG10 CCT2 CAGCTTCCT1	5	0.51%	0	0.00%	0	0.00%	0	0.00%

Table S2. Synthetic ultramer sequences used to assess polymorphisms near the CAG repeat tract. "C" designates canonical single CAA interruption variations and "NC" non-canonical variations as defined in Table S1 of GeM-HD Consortium et al., Cell (2019): 178, 887-900. The most common six canonical and four non-canonical polymorphisms (Table S1) were selected for evaluation using the AmplideX PCR/CE *HTT* Kit.

T15777_19CAG_C1
GGCCCGAGGCCTCCGGGGACTGCCGTGCCGGGCGGGAGACCGCCATGGCGACCCTGGAAAAGCTGATGAAG
GAATTCGAGTCCCTCAAGTCCTTCCAGCAGCAGCAGCAGCAGCAGCAGCAGCAGCAGCAGCAGCAGCAGC
AGCAGCAGCAACAGCCGCCACCGCCGCCGCCGCCGCCGCCTCCTCAGCTTCCT

T15778_19CAG_C2
CCTCCGGGGACTGCCGTGCCGGGCGGGAGACCGCCATGGCGACCCTGGAAAAGCTGATGAAGGAATTCGAGT
CCCTCAAGTCCTTCCAGCAGCAGCAGCAGCAGCAGCAGCAGCAGCAGCAGCAGCAGCAGCAGCAGCAGC
AACAGCCGCCACCGCCGCCGCCGCCGCCGCCGCCGCCGCCTCCTCAGCTTCCT

T15779_19CAG_C3
GGCCCGAGGCCTCCGGGGACTGCCGTGCCGGGCGGGAGACCGCCATGGCGACCCTGGAAAAGCTGATGAAG
GAATTCGAGTCCCTCAAGTCCTTCCAGCAGCAGCAGCAGCAGCAGCAGCAGCAGCAGCAGCAGCAGCAGC
AGCAGCAGCAACAGCCGCCGCCGCCGCCGCCGCCGCCGCCTCCTCAGCTTCCT

T15780_19CAG_C4
AGGCCTCCGGGGACTGCCGTGCCGGGCGGGAGACCGCCATGGCGACCCTGGAAAAGCTGATGAAGGAATTCG
AGTCCCTCAAGTCCTTCCAGCAGCAGCAGCAGCAGCAGCAGCAGCAGCAGCAGCAGCAGCAGCAGCAGC
AGCAACAGCCGCCACCGCCGCCGCCGCCGCCGCCGCCGCCTCCTCAGCTTCCT

T15781_19CAG_C5
CCTCCGGGGACTGCCGTGCCGGGCGGGAGACCGCCATGGCGACCCTGGAAAAGCTGATGAAGGAATTCGAGT
CCCTCAAGTCCTTCCAGCAGCAGCAGCAGCAGCAGCAGCAGCAGCAGCAGCAGCAGCAGCAGCAGCAGCAGC
AACAGCCGCCACCGCCGCCGCCGCCGCCGCCGCCGCCTCCTCCTCAGCTTCCT

T15782_19CAG_C6
GTCGGCCCGAGGCCTCCGGGGACTGCCGTGCCGGGCGGGAGACCGCCATGGCGACCCTGGAAAAGCTGATGA
AGGAATTCGAGTCCCTCAAGTCCTTCCAGCAGCAGCAGCAGCAGCAGCAGCAGCAGCAGCAGCAGCAGCAGCA
GCAGCAGCAGCAACAGCCGCCACCGCCGCCGCCGCCGCCTCCTCAGCTTCCT

T15783_19CAG_NC1
CCTCCGGGGACTGCCGTGCCGGGCGGGAGACCGCCATGGCGACCCTGGAAAAGCTGATGAAGGAATTCGAGT
CCCTCAAGTCCTTCCAGCAGCAGCAGCAGCAGCAGCAGCAGCAGCAGCAGCAGCAGCAGCAGCAGCAGCAGC
AACAGCAACAGCCGCCACCGCCGCCGCCGCCGCCGCCTCCTCCTCAGCTTCCT

T15784_19CAG_NC2
CGAGTCGGCCCGAGGCCTCCGGGGACTGCCGTGCCGGGCGGGAGACCGCCATGGCGACCCTGGAAAAGCTGA
TGAAGGAATTCGAGTCCCTCAAGTCCTTCCAGCAGCAGCAGCAGCAGCAGCAGCAGCAGCAGCAGCAGCAGCA
GCAGCAGCAGCAGCCGCCACCGCCGCCGCCGCCGCCGCCTCCTCAGCTTCCT

15785_19CAG_NC3
CCGAGGCCTCCGGGGACTGCCGTGCCGGGCGGGAGACCGCCATGGCGACCCTGGAAAAGCTGATGAAGGAAT
TCGAGTCCCTCAAGTCCTTCCAGCAGCAGCAGCAGCAGCAGCAGCAGCAGCAGCAGCAGCAGCAGCAGCAGCA
GCAGCCGCCGCCGCCGCCGCCGCCGCCGCCGCCGCCGCCTCCTCAGCTTCCT

T15786_19CAG_NC4
GTCGGCCCGAGGCCTCCGGGGACTGCCGTGCCGGGCGGGAGACCGCCATGGCGACCCTGGAAAAGCTGATGA
AGGAATTCGAGTCCCTCAAGTCCTTCCAGCAGCAGCAGCAGCAGCAGCAGCAGCAGCAGCAGCAGCAGCAGCA
GCAGCAGCAGCCGCCGCCGCCGCCGCCGCCGCCGCCGCCTCCTCAGCTTCCT

Figure S1: Electropherograms demonstrating amplification and genotyping of synthetic ultramer DNA templates with canonical (A-F) and non-canonical (G-J) sequence variations detailed in Tables S1 and S2. The reference (control) electropherogram is shown in panel K. DNA templates were input into *HTT* PCR at 10,000 copies, comparable to the haploid copy number of the *HTT* gene in 30 ng of genomic DNA.

A) T15777_19CAG_C1: *HTT* Genotype 19 CAGs

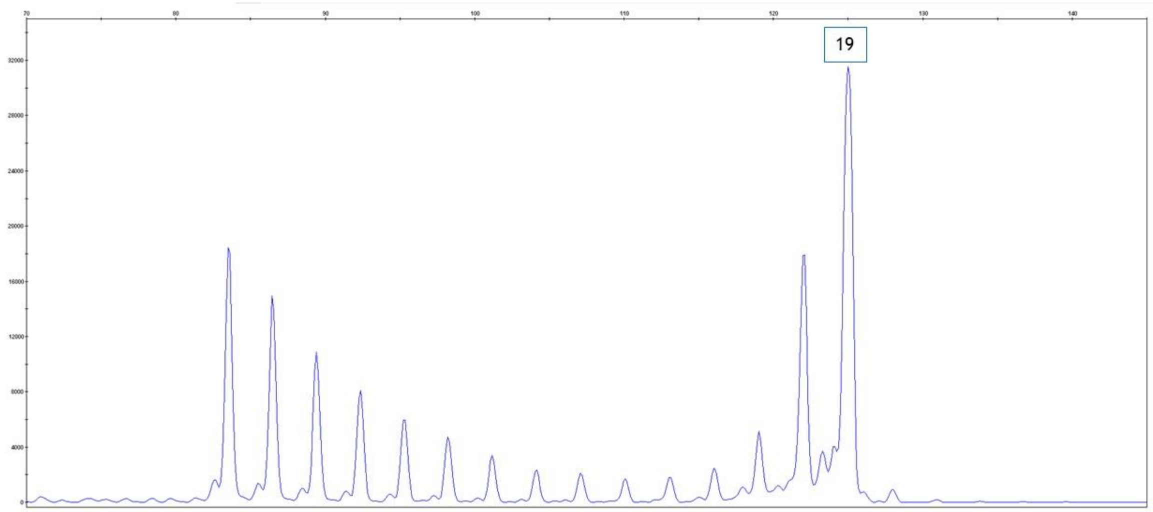

B) T15778_19CAG_C2: *HTT* Genotype 19 CAGs

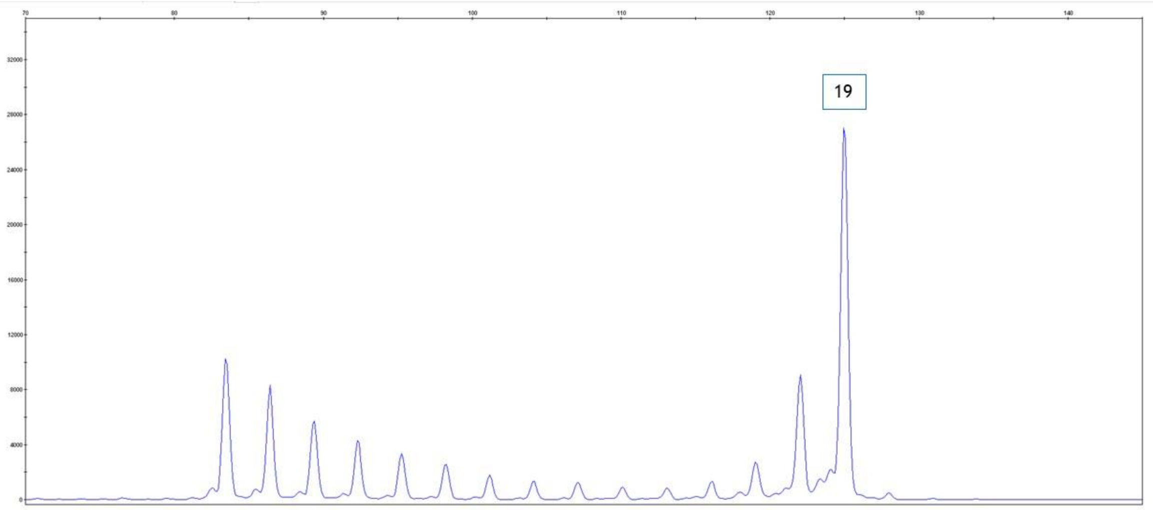

C) T15779_19CAG_C3: *HTT* Genotype 19 CAGs

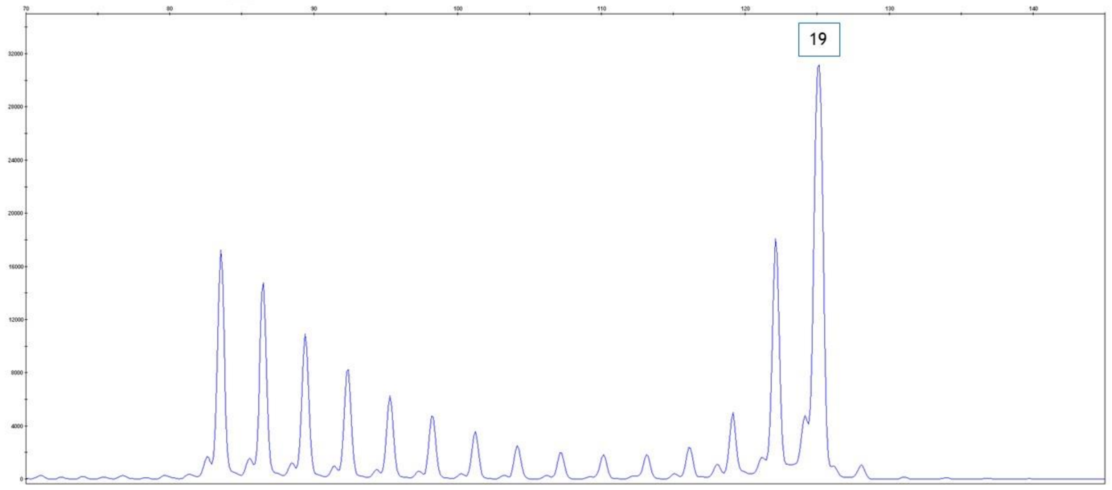

D) T15780_19CAG_C4: *HTT* Genotype 19 CAGs

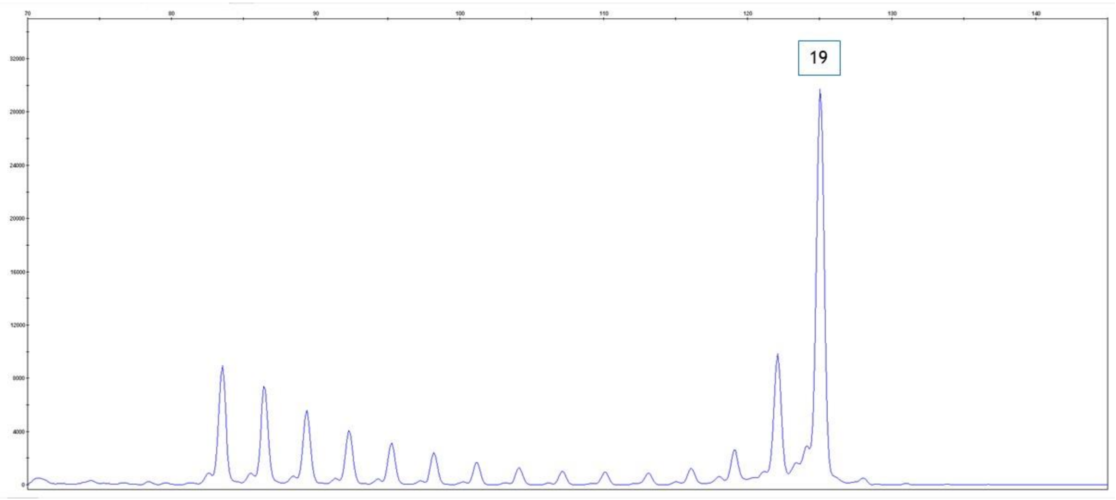

E) T15781_19CAG_C5: *HTT* Genotype 19 CAGs

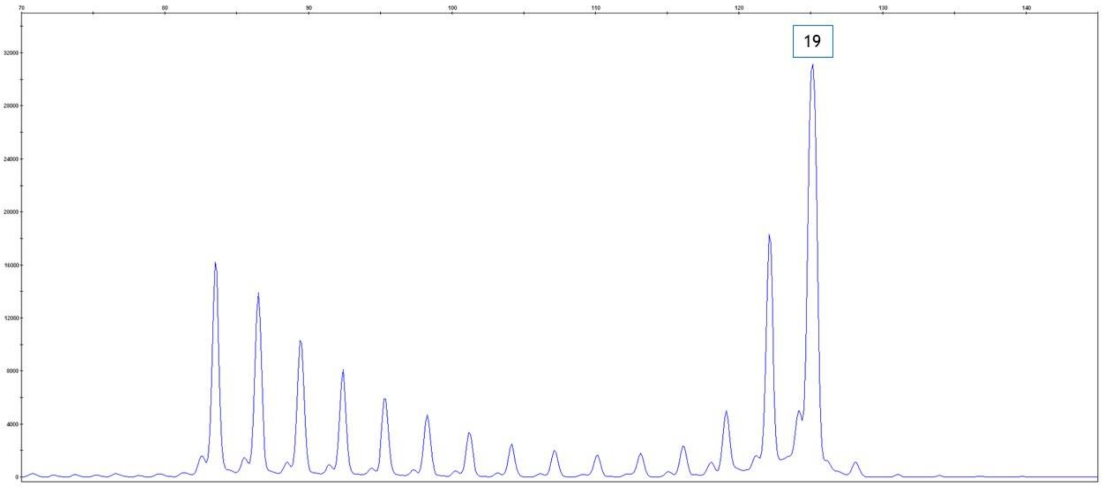

F) T15782_19CAG_C6: *HTT* Genotype 19 CAGs

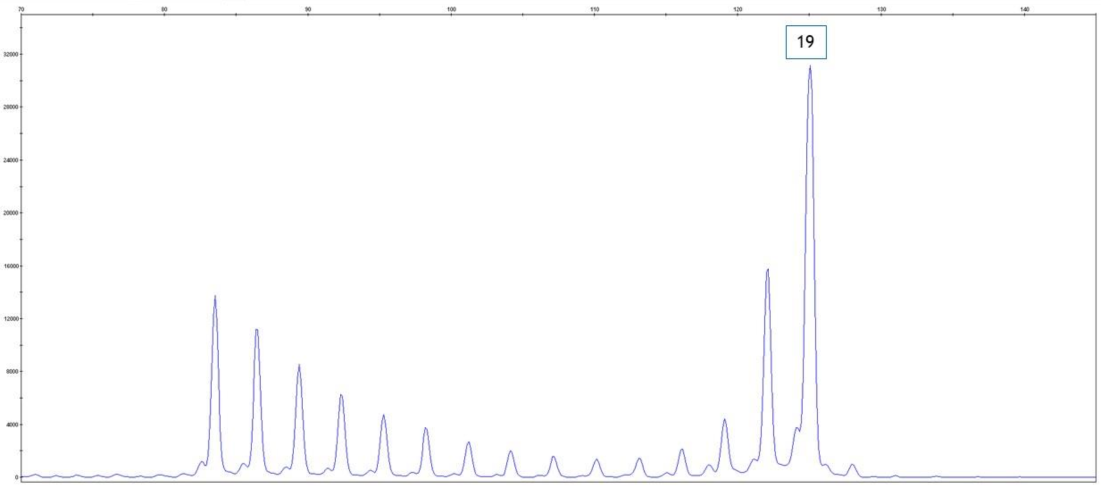

G) T15783_19CAG_NC1: *HTT* Genotype 20 CAGs

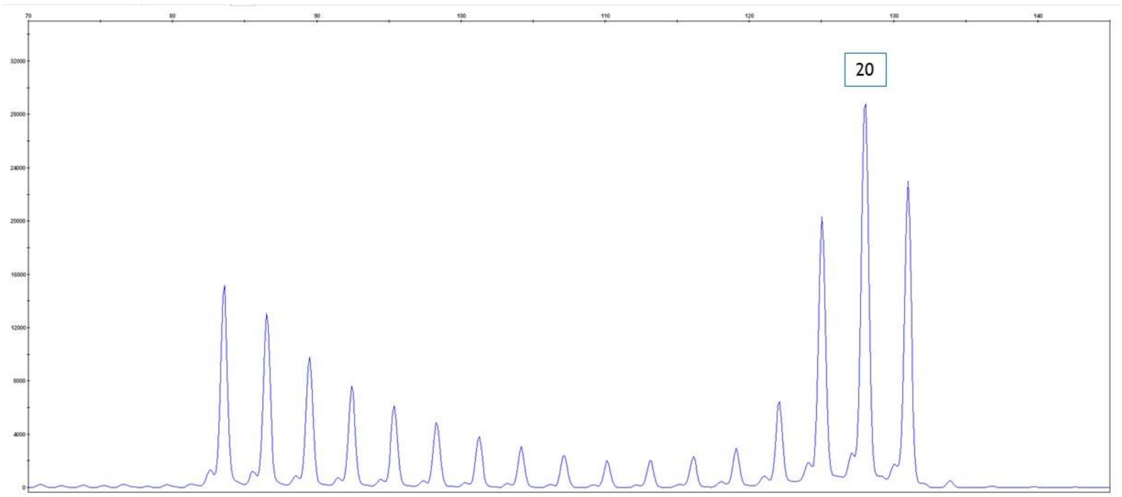

H) T15784_19CAG_NC2: *HTT* Genotype 17 CAGs, shifted due to missing spacer sequence

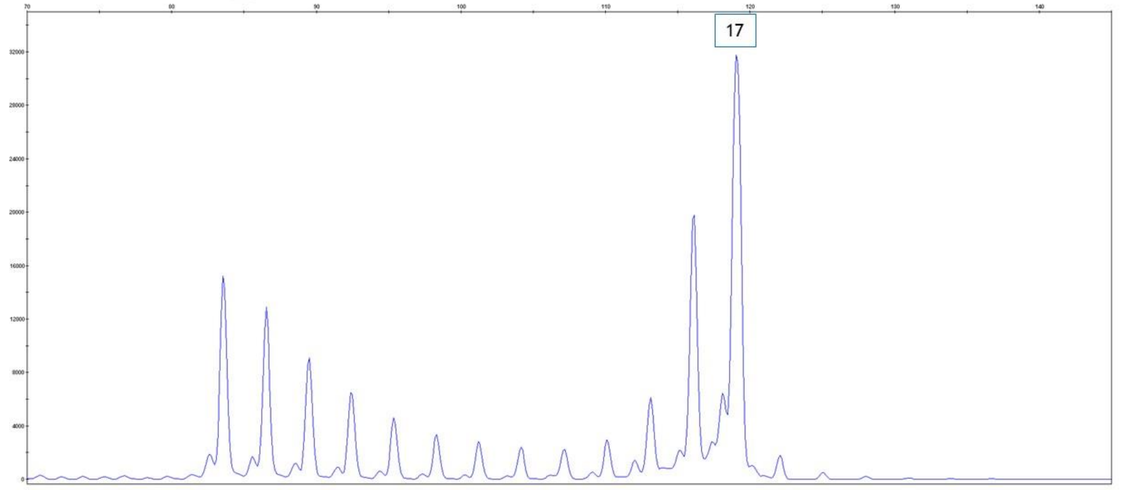

I) 15785_19CAG_NC3: *HTT* Genotype 17 CAGs, shifted due to missing spacer sequence

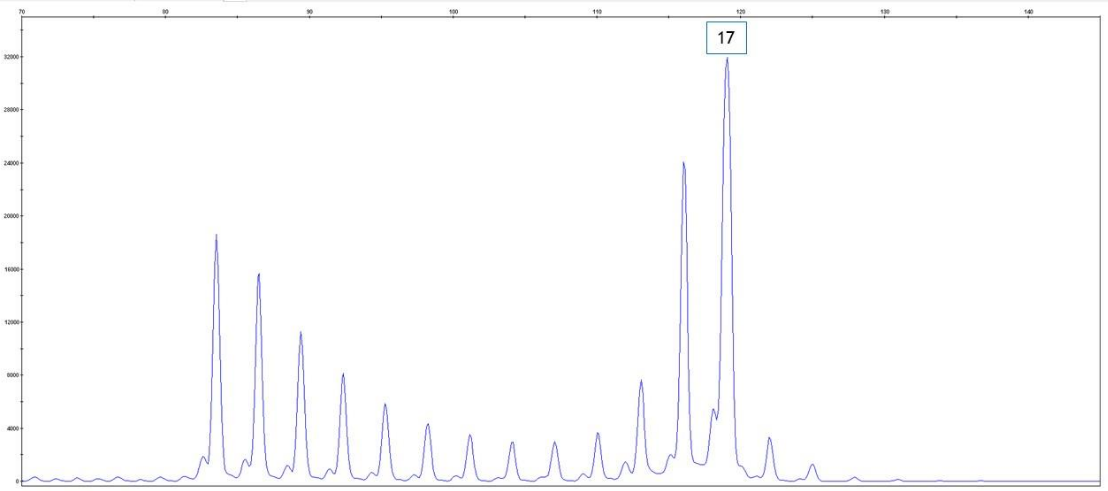

J) T15786_19CAG_NC4: *HTT* Genotype 17 CAGs, shifted due to missing spacer sequence

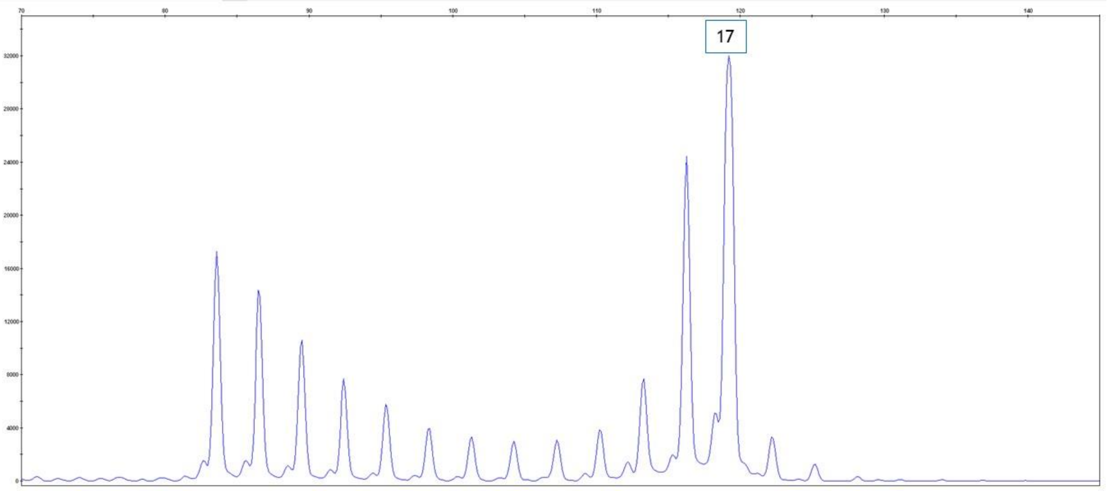

K) T15787_*HTT*_19CAG7CCG, Reference (control) sequence: *HTT* Genotype 19 CAGs

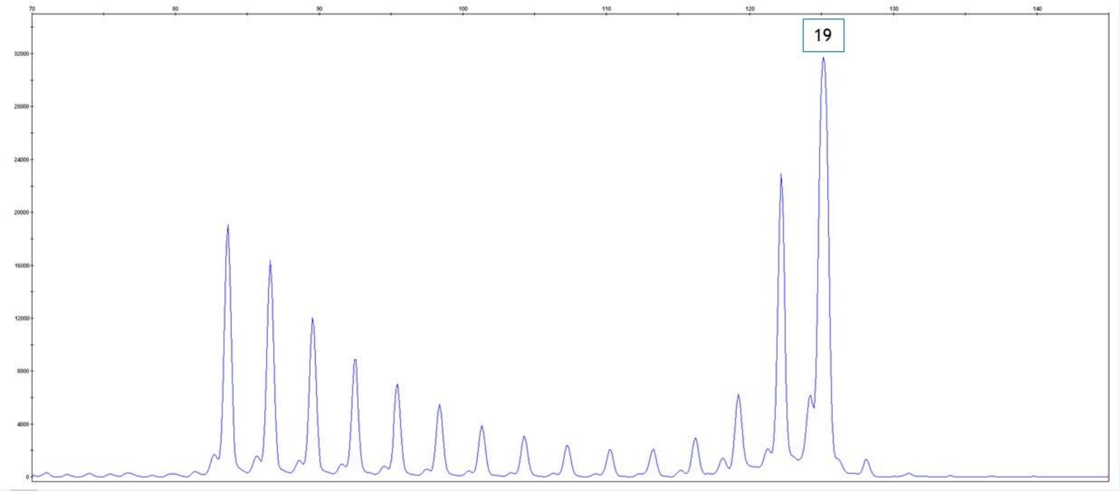

Supplement: Supplementary file 1 [file ijms-22-01689-s001.pdf]
